# An Atypical Presentation of Dulaglutide-Induced Pancreatitis Complicated by Superior Mesenteric Vein Thrombosis

**DOI:** 10.7759/cureus.50051

**Published:** 2023-12-06

**Authors:** Sebastian L Manuel, Frank Lin, Sinan M Kutty

**Affiliations:** 1 Internal Medicine, St. Luke's Hospital, Easton, USA; 2 Gastroenterology, St. Luke's Hospital, Easton, USA

**Keywords:** abdominal pain, pancreatic mass, superior mesenteric vein thrombosis, pancreatitis, glp-1 agonist

## Abstract

Glucagon-like peptide 1 (GLP-1) agonists are commonly used in the management of type 2 diabetes due to their glucose-lowering effects and potential cardiovascular benefits. While generally well-tolerated, here we report a unique case associated with GLP-1 therapy. A 57-year-old male with a history of type 2 diabetes developed mild abdominal pain with no nausea or vomiting approximately four months after increasing the dose of GLP-1 therapy. Imaging studies revealed mesenteric vein thrombosis and an enlarged pancreatic head. Endoscopic ultrasound with biopsies was completed, which confirmed acute pancreatitis. The patient was promptly treated with a heparin drip and supportive care. The GLP-1 agonist was discontinued. This case highlights a rare but critical adverse event associated with GLP-1 receptor agonists as well as the importance of considering unusual complications in patients initiating such therapy. Further research is warranted to elucidate the underlying mechanisms and risk factors for these adverse events.

## Introduction

The glucagon-like peptide 1 (GLP-1) agonists have emerged as a cornerstone treatment option for type 2 diabetes mellitus. Their use has increased due to their association with weight loss and cardiovascular benefits [1]. The drug class is generally safe; however, numerous studies have linked it with pancreatitis [2-7]. We present a unique case of a patient who was found to have mesenteric vein thrombosis and subacute pancreatitis after increasing his dose of dulaglutide.

## Case presentation

This case report presents a 57-year-old male of Southeast Asian descent admitted to the hospital due to abdominal pain. The patient first had abdominal pain two weeks prior, which he described as diffuse, worse in the substernal/epigastric region, and on the right upper side of the abdomen. The pain did not radiate and was persistent, with a severity of 3/10. At that time, the patient thought he had "gas." The pain was not relieved with over-the-counter antacids. He did not have changes to his bowel movement. He denied nausea and vomiting. The day prior to admission, the pain worsened to eight out of 10 and he felt subjectively feverish. This ultimately prompted the patient to go to the emergency department, where he was subsequently admitted to the hospital.

The patient denied illicit drug or alcohol use. He was a former smoker with a 10-pack-year history and quit four years prior. One week before admission, he went on a road trip by car that was around eight hours long. He denied a history of deep vein thrombosis or pulmonary embolism. He had no family history of clotting and no family history of cancer. He did not have a history of gallstones. 

His medical history was significant for diabetes, for which he was taking metformin 1,000 mg two times a day, dulaglutide 3 mg/0.5 mL every seven days for about two years, dapagliflozin 10 mg tablet once daily, insulin aspart 12 units three times a day before meals, and insulin glargine 44 units under the skin once daily. He had had good diabetes control with glycated hemoglobin (Hba1c) <7% for the past few years. Three months before admission, the patient's dulaglutide was increased from 1.5 mg every seven days to 3 mg every seven days to help weight loss and reduce insulin requirements.

Of note, he was also on allopurinol 200 mg once daily for his gout and atorvastatin 40 mg once daily for hyperlipidemia. He was on amlodipine 10 mg once daily, lisinopril 20 mg once daily, and metoprolol tartrate 50 mg two times a day for his hypertension. He also had been taking pantoprazole 40 mg once daily for gastrointestinal reflux disease. He had a medical history of achalasia, a status post Heller myotomy that was complicated by an esophageal perforation and repaired with Dor fundoplication a few months prior.

In the hospital, the patient was afebrile and had stable vital signs. On physical examination, the patient had abdominal tenderness in the right upper quadrant. There were no noted rashes. No peripheral edema was noted. No rebound tenderness was noted. All other parts of the physical exam were unremarkable.

**Figure 1:**
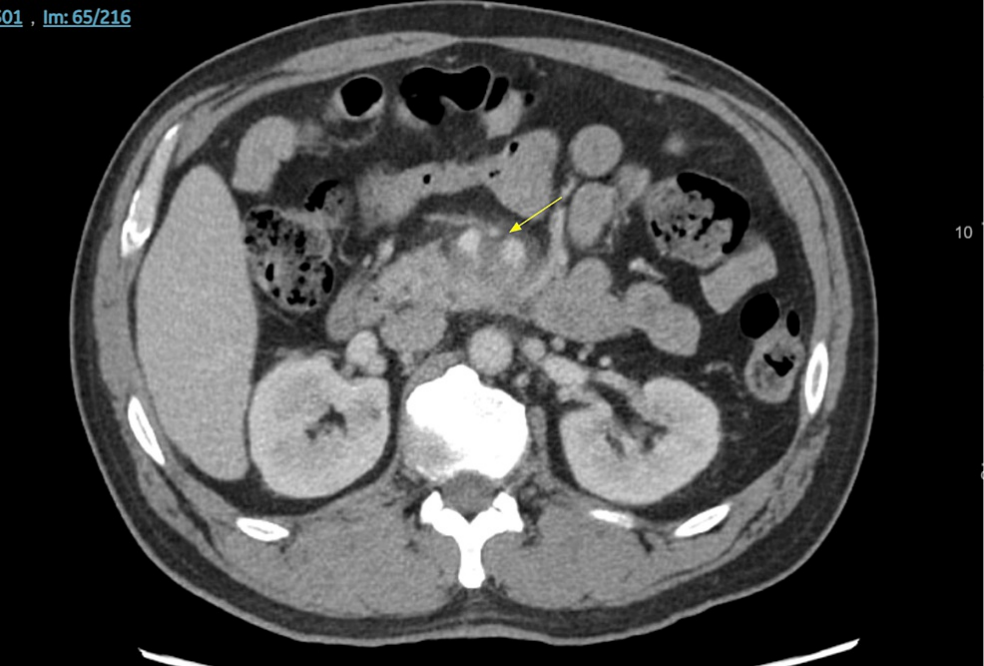
A CT scan of the abdomen and pelvis with contrast shows occlusive thrombosis involving the proximal jejunal vein. A hazy inflammatory stranding was noted abutting the pancreatic uncinate process, which raised concern for focal acute pancreatitis or infiltrative pancreatic neoplasm. The yellow arrow points at where the proximal jejunal vein abruptly stops before draining into the superior mesenteric vein. Directly posterior to this point, the previously mentioned hazy inflammatory stranding is visualized.

A CT scan of the abdomen and pelvis with contrast was done, which showed findings suspicious for focal occlusive thrombosis involving the proximal jejunal vein (the proximal branch of the superior mesenteric vein). A hazy inflammatory stranding was noted around the pancreatic uncinate process, which raised concern for focal acute pancreatitis or infiltrative pancreatic neoplasm (Figure 1).

The portal and splenic veins were grossly patent and unremarkable. No gallstones were noted.

A mesenteric duplex was done, which showed >70% stenosis at the origin of the superior mesenteric artery. The superior mesenteric artery appeared aneurysmal/ectatic and measured 1.13 cm at its largest diameter.

Lipase was noted to be marginally elevated at 101 u/L (normal range: 11-82 u/L). The white blood cell (WBC) count was also marginally elevated at 10.50 thousand/uL. Lactic acid was normal at 0.6 mmol/L. The basic metabolic panel (BMP) was unremarkable. Creatinine was at 1.6 mg/dl (the baseline was 1.8-1.9). A coagulation panel was ordered due to the superior mesenteric vein thrombosis found on the CT scan. Prothrombin time (PT), international normalized ratio (INR), and partial thromboplastin time (PTT) were normal. Protein S and protein C were also within normal levels. Antithrombin 3 activity was slightly decreased at 89% (compared to normal at 92%-136%). In the emergency room, the patient was given 1 liter of normal saline bolus, and he was maintained at 100 mL/hr while admitted.

**Figure 2:**
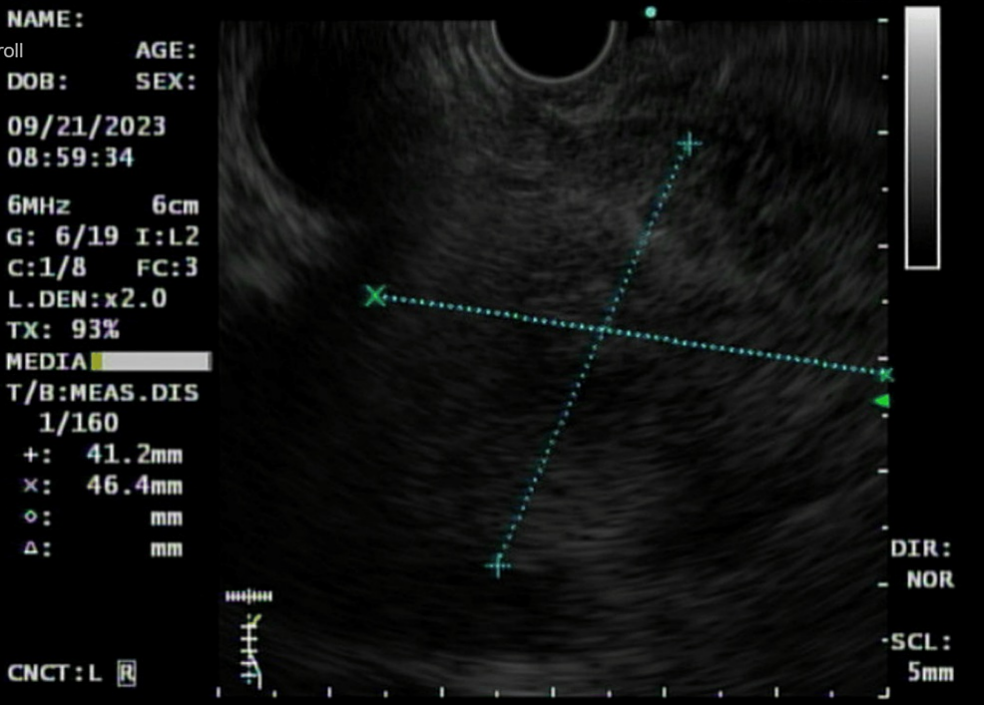
Endoscopic ultrasound findings of a 4.5-centimeter fullness in the head of the pancreas are isoechoic to the remainder of the pancreas.

The gastroenterology and vascular surgery consulting teams were consulted. The patient was started on a heparin drip. The vascular consulting team advised continuing anticoagulation for six months and recommended no surgical intervention. A GI-recommended endoscopic ultrasound (EUS), which showed a 4.5-centimeter fullness in the head of the pancreas, was isoechoic compared to the rest of the pancreas (Figure 2).

The pancreatic duct tapered smoothly towards the ampulla. Fine needle aspiration was performed using a 22- and 25-gauge needle. Reactive lymph nodes were seen in the peripancreatic region. The remainder of the pancreas body and tail appeared normal. A normal left lobe of the liver was noted. The normal celiac artery, superior mesenteric artery, and portal triad were noted. The biopsy results showed atypical pancreatic acinar cells with mild cytologic atypia, which favors reactive changes. Neutrophils, histiocytes, and lymphocytes were noted in the pathology report. Necrotic debris was also noted in the pathology report.

After the endoscopic ultrasound and biopsies, his pancreatitis worsened. His serum lipase further increased to 3,499 u/L at that time. He was treated with fluids and pain management. After a few more days, the patient showed improvement, and he was sent home with apixaban to complete for six months. After a discussion with his endocrinologist, he was advised to discontinue dulaglutide after discharge. Follow-up was arranged with GI and vascular services after discharge. Due to the mass noted on the uncinate process of the pancreas, the patient was advised to have a repeat CT angiogram of the abdomen and pelvis six weeks after discharge, which showed an ill-defined hypovascular area corresponding with the sequela of acute pancreatitis.

## Discussion

Patients with acute pancreatitis typically present with the cardinal symptom of abdominal pain. The pain is usually localized to the epigastrium, right upper quadrant, and occasionally the left upper quadrant with radiation in a bandlike manner to the lower thoracic region [2].

The diagnosis is often made with two of the following: characteristic abdominal pain, elevation in serum lipase or amylase to three times or greater than the upper limit of normal, or characteristic findings of acute pancreatitis on imaging (contrast-enhanced computed tomography, magnetic resonance imaging, or transabdominal ultrasonography) [3].

The patient in our case did not meet this criteria. He presented with mild abdominal pain and lipase levels that were only marginally elevated at 101 u/L (normal 5-60 u/L) and did not meet the criteria of three times the upper limit of normal. The patient also had no history of alcoholism, no evidence of gallstones on the CT scan, and normal triglycerides, which are other common causes of acute pancreatitis [2].

Our patient had recently increased his dosage of dulaglutide, a member of the GLP-1 agonist drug class. The GLP-1 agonists were associated with an increased risk for hospitalization for acute pancreatitis, with an odds ratio of 2.07 and a 95% confidence interval [4]. Other population-based cohort studies, however, noted the incidence to be low (16 cases among 14,562 patients enrolled in GLP-1 receptor agonist randomized trials) [5]. Some literature describes GLP-1-induced chronic pancreatitis and subclinical pancreatic inflammation [6-7]. In some trials, pancreatitis associated with GLP-1 agonists had normal amylase and lipase levels [8]. Based on the literature review, current use (GLP-1 agonist started less than 30 days prior) and recent use (GLP-1 agonist started from 30 days to two years prior) were associated with statistically significantly higher odds of acute pancreatitis [4]. This timeframe seems to fit our patient, who had been taking dulaglutide for two years and recently increased the dose three months before being hospitalized.

This case is also unique because the patient was found to have mesenteric vein thrombosis.

The finding of isolated thrombosis involving the superior mesenteric vein is very rare and is usually seen with intra-abdominal sepsis or pancreatic neoplasm. The incidence of isolated superior mesenteric vein involvement is reported to be 5%-15% in all cases of mesenteric vessel occlusive disease [9].

The most common etiologies of mesenteric venous thrombosis are prothrombotic states due to heritable or acquired disorders of coagulation or cancer, intra-abdominal inflammatory conditions such as pancreatitis, the postoperative state, and cirrhosis and portal hypertension [9-11].

The cause of mesenteric venous thrombosis involving the superior mesenteric vein in our patient was suspected to be secondary to acute pancreatitis induced by dulaglutide. The patient was started on long-term anticoagulation with apixaban on discharge. Treatment for mesenteric venous thrombosis includes either medical management or surgical intervention in the form of a thrombectomy. Mesenteric venous thrombosis can be safely managed without surgery if there is no evidence of a bowel infarction. Immediate anticoagulation with heparin early in the disease course increases survival and significantly decreases the risk of recurrence [9-10]. The patient should be anticoagulated for three to six months. Warfarin with an INR ratio of 2-3 can be used. Direct thrombin and factor Xa inhibitors can also be used [9-11]. The patient was initially started on a heparin drip and sent home with apixaban.

## Conclusions

In conclusion, this was a unique case of mesenteric vein thrombosis and pancreatitis after increasing the dosage of dulaglutide. Hopefully, this case report was able to shed light on possible complications that can emerge from using GLP-1 receptor agonists. While the benefits of GLP-1 receptor agonists are becoming more familiar and their safety profile is generally favorable, healthcare providers must remain vigilant in assessing potential risks. The rarity of mesenteric vein thrombosis upon initiating this drug class warrants further investigation to elucidate the underlying mechanisms and identify potential risk factors.
